# The impact of computerized agility training on basketball performance: a comparative study with rope ladder training

**DOI:** 10.3389/fphys.2026.1772554

**Published:** 2026-02-17

**Authors:** Mingquan Zhang, Wenlong Zhang, Rui Chen, Yana Liu, Jorge Diaz-Cidoncha Garcia, Chao Chen, Xiao Xu

**Affiliations:** 1 College of Physical Education, Dalian University, Dalian, Liaoning, China; 2 Fédération Internationale de Football Association (FIFA), Zurich, Switzerland

**Keywords:** linear mixed-effects models, position-specific adaptations, pre-planned agility, QuickBoard, reactive agility, training intervention

## Abstract

**Background:**

Agility is a critical determinant of basketball performance, enabling rapid directional changes, movement reorganization, and real-time decision-making under competitive pressure.

**Objectives:**

To evaluate the effects of a four-week computerized agility training (CAT) program and rope ladder training (RLT) on general agility and basketball-specific skill performance in collegiate male basketball players. The study also aimed to examine position-specific training responses and quantify individual variability using linear mixed-effects models (LMM).

**Methods:**

In a randomized controlled trial, 64 male collegiate basketball players (aged 18–24; guards = 26, forwards = 26, centers = 12) were randomly assigned to either the CAT or RLT groups (n = 32 each group). Both interventions were performed three times per week for 4 weeks. Pre- and post-intervention assessments included a footwork speed test, a T-test for change of direction, and measures of choice reaction time and accuracy. Basketball-specific proficiency was assessed using the Combined Basketball Skill Test (CBST), incorporating performance time, penalty time, and stimulus response time. LMM were used to analyze the training effects.

**Results:**

Compared with RLT, CAT elicited significantly greater improvements in foot speed (+7.0% vs. +2.4%), choice reaction time (−6.9% vs. −0.7%), and CBST reaction time (−9.8% vs. −1.4%) (p < 0.05). CAT also led to larger reductions in overall CBST performance time (−2.1% vs. −0.3%) and completion time (−2.3% vs. −0.5%), although penalty time decreased significantly only among centers. Positional effects were observed: guards exhibited superior baseline agility, while centers demonstrated greater adaptive gains in decision-making and directional change. LMM revealed small to large effect sizes (Cohen’s f = 0.12–0.74) and high conditional *R* ^2^ values (0.71–0.95), reflecting robust model fit and substantial inter-individual variability.

**Conclusion:**

Computerized agility training significantly enhanced reactive agility and basketball-specific skill execution beyond rope ladder training, particularly in tasks involving perception and decision-making. The effects were position-dependent, with centers benefiting most from CAT. These findings highlight the limitations of traditional footwork drills and support the integration of perception–action–coupled, individualized agility training paradigms. Future research should explore the neurophysiological mechanisms and long-term retention of CAT in basketball conditioning.

## 1 Introduction

Basketball is a high-intensity sport that requires rapid movements, abrupt stops, cutting actions, and complex ball-handling skills (Stojanović et al., 2018; Versic et al., 2021; Cui et al., 2019; Abdelkrim et al., 2010). High-level performance relies heavily on an athlete’s ability to accelerate, decelerate, and make quick decisions under time constraints (Fort-Vanmeerhaeghe et al., 2016; Hoare, 2000). As the physical demands in elite basketball intensify, agility has become a critical determinant of performance (Hassan et al., 2022; Gray and Jenkins, 2010; Barnes et al., 2014). Unlike linear speed or strength, agility involves a complex interaction of perceptual, cognitive, and motor processes (Lloyd et al., 2015; Scanlan et al., 2014). Elite athletes demonstrate faster tactical responses and the ability to adapt their movement strategies instantaneously (Young et al., 2011; Serpell et al., 2011; Sun et al., 2025). Superior agility enables players to handle unpredictable in-game situations, respond quickly to cues, and optimize spatial positioning (Delextrat and Cohen, 2009). Therefore, developing sport-specific agility is essential for improving basketball performance (Wang et al., 2024).

Agility is the ability to rapidly adjust movement patterns or direction in response to task demands (Chong et al., 2021). While its definition varies, it is generally understood as comprising three components: (a) change of direction ability, (b) action transformation ability, and (c) reaction and decision-making ability (Zhao et al., 2015). Recent agility training emphasizes not only footwork and speed but also perceptual-cognitive components, including reaction time, anticipatory judgment, and movement decision-making (Casamento-Moran et al., 2019; Tarkka and Hautasaari, 2019; Trofimova et al., 2020; Nascimento et al., 2020; Presta et al., 2021; Afonso et al., 2020; Pradana et al., 2020; Liu, 2023). In basketball, two primary training methods are used: Rope Ladder Training (RLT), which focuses on rhythm and coordination through predefined patterns, and Computerized Agility Training (CAT), which incorporates randomized visual stimuli and reactive tasks (Jorgensen et al., 2013; Paul et al., 2016; Galpin et al., 2008; Engeroff et al., 2019; Collins, 2014; Engeroff et al., 2018; Engeroff et al., 2021). Although RLT is widely used due to its low cost, its lack of ecological validity limits its ability to simulate real-game conditions (Ng et al., 2017; Bassa et al., 2024; Padrón-Cabo et al., 2021; Smits-Engelsman et al., 2019). In contrast, CAT uses randomized cues to enhance perceptual-motor integration and decision-making (Engeroff et al., 2021).

Despite growing interest in perceptual-cognitive agility, few studies have directly compared CAT and RLT in basketball populations. Existing research often relies on generalized agility protocols rather than basketball-specific drills that integrate decision-making with technical execution. Additionally, basketball positions (guards, forwards, and centers) have distinct agility profiles, yet position-specific responses to training remain underexplored (Kutlu and Dogan, 2018; Stojanović et al., 2019). Moreover, previous studies have overlooked individual variability and the nested structure of training data, limiting the generalizability of their conclusions. Rigorous experimental designs that assess the differential impacts of CAT and RLT on agility and basketball-specific performance remain scarce.

This study addresses the gaps in existing research by conducting a 4-week randomized controlled trial comparing CAT and RLT in male collegiate basketball players. The effects on action transformation, change of direction, reaction and decision-making performance, and basketball-specific skills were assessed. The training protocol was designed to replicate basketball’s dynamic perceptual-motor demands.

In addition, three key advancements are introduced: (a) the integration of decision-making and movement coupling in a basketball-specific agility test, enhancing ecological validity; (b) the use of linear mixed-effects models to account for individual variability, thereby strengthening inferential robustness; and (c) a comparative analysis of position-specific training responses, which supports individualized training prescriptions. The study aims to provide evidence-based guidance for basketball periodization, position-specific physical preparation, and precision training strategies for agility development in fast-paced, high-intensity team sports.

## 2 Materials and methods

### 2.1 Participants

This study involved collegiate student-athletes, and institutional approval was obtained from the College of Physical Education at Dalian University for participant enrollment. Prior to enrollment, all prospective participants were briefed on the study protocol, including intervention procedures and testing schedules. Inclusion criteria were as follows: (a) National second-level basketball athlete in China; (b) at least 3 years of systematic basketball training since high school; (c) experience as a primary rotation player in provincial or higher-level competitions; (d) medically cleared and physically capable of completing all assessments; (e) right-hand dominance; (f) no neuromuscular or movement-related injuries within the last 6 months; and (g) normal vision, with no color blindness or color weakness.

The imbalance in the number of athletes across positions reflects the structural characteristics of basketball rosters. Although the *a priori* power calculation was based on the total sample size, smaller positional subsamples—particularly for centers—may reduce the precision of position-specific estimates. To account for the unbalanced positional structure and repeated measurements, linear mixed-effects models (LMM) were used, which provide robust inference under unequal group sizes and allow positional effects to be modeled while appropriately handling within-subject dependence (Verbeke, 2000).

A total of 64 male basketball players (aged 18–24 years) volunteered to participate and were randomly assigned to either the computerized agility training group (CAT; n = 32; Centers = 6; Forwards = 13; Guards = 13) or the rope ladder agility training group (RLT; n = 32; Centers = 6; Forwards = 13; Guards = 13). Group characteristics are provided in Table 1.

**TABLE 1:** Baseline demographic and training characteristics of participants by group and playing position.

Variable	CAT				RLT			
	All positions (n = 32)	Guards (n = 13)	Forwards (n = 13)	Centers (n = 6)	All positions (n = 32)	Guards (n = 13)	Forwards (n = 13)	Centers (n = 6)
Age (years)	21.31 ±1.55	20.92 ±1.25	21.31 ±1.85	22.50 ±0.55	21.81 ±1.55	21.38 ±1.56	22.31 ±1.24	21.83 ±2.14
Height (cm)	189.13 ±6.45	184.92 ±4.12	189.15 ±4.52	198.17 ±2.04	186.31 ±5.79	181.92 ±3.11	188.15 ±2.02	194.67 ±2.58
Weight (kg)	87.44 ±9.66	80.92 ± 5.21	87.31 ± 5.85	103.00 ± 3.69	84.97 ±7.46	79.31 ± 5.00	85.77 ± 3.68	95.50 ± 7.18
BMI (kg/m^2^)	24.37 ±1.40	23.83 ± 1.29	24.27 ± 0.97	26.23 ± 0.78	24.45 ±1.33	24.42 ± 1.29	24.17 ± 1.17	25.19 ± 1.64
Training experience (years)	6.13 ±1.07	6.54 ±0.87	5.38 ±0.85	6.83 ±0.75	5.19 ±0.93	4.85 ±0.99	5.31 ±0.88	5.33 ±0.82
HRrest (bpm)	58.41 ±2.78	57.00 ± 2.55	58.46 ± 2.18	61.33 ± 2.34	58.28 ±2.65	57.31 ± 2.29	58.00 ± 2.42	61.00 ± 2.37
HRmax (bpm)	192.62 ± 1.04	193.04 ± 0.84	192.57 ± 1.24	191.83 ± 0.37	192.29 ± 1.04	192.57 ± 1.04	192.01 ± 0.83	192.27 ± 1.43

Values are presented as mean ± SD., CAT, computerized agility training; RLT, rope ladder training; BMI, body mass index; HRrest, Resting Heart Rate; HRmax, Maximal Heart Rate.

Written informed consent was obtained from all participants before enrollment. The study adhered to the Declaration of Helsinki and was approved by the institutional ethics committee (Approval No. DLUSPR20250506). All data were kept confidential and used exclusively for research purposes.

### 2.2 Experimental design

Rope ladder training (RLT) is a widely used basketball agility drill. Both CAT and RLT involve similar multi-directional footwork with overlapping patterns, stride frequency, and length, but they organize the same repertoire differently. Both methods are short, high-frequency, intermittent drills designed for rapid footwork and neural activation, but they employ distinct sensory stimuli. These drills simulate game actions such as defensive shuffling, help-defense rotations, and post-drive adjustments.

This study adopted a mixed (Group × Time) design to compare the effects of computerized agility training (CAT) and rope ladder training (RLT) on agility and basketball-specific performance. The trial was conducted in the basketball arena of the School of Physical Education, Dalian University, from May to July 2025. Each group completed a 4-week intervention, with three sessions per week and at least 24 h between sessions. Random allocation was used to reduce selection bias. To limit expectancy effects, participants were informed that both interventions were commonly used agility approaches and were not told the specific study hypotheses. All testing and training sessions were supervised by the principal investigator, and pre- and post-intervention assessments were administered at the same time of day to ensure consistency.

In the week prior to baseline testing, participants underwent a 1-week familiarization period (three sessions) to learn the testing procedures, intervention drills, and the use of the Firstbeat professional heart-rate monitor (Finland; sensor: MOVESENSE; model OP174; product ID 213830002517). This familiarization period was implemented to reduce learning and novelty-related effects associated with the computerized equipment and testing procedures. Agility exercises, basketball-specific drills, and test sequences were introduced under coach-led technical instruction, with oversight provided by a strength-and-conditioning specialist. The training plans are outlined in Tables 2, 3. Following familiarization, participants were randomly assigned to either the computerized agility training group (CAT, n = 32; centers = 6, forwards = 13, guards = 13) or the rope ladder training group (RLT, n = 32; centers = 6, forwards = 13, guards = 13) using the SPSS random number generator.

**TABLE 2:** Training content for CAT.

Week	Type	Content	Duration (min)	Set (n)	Inter-drill rest (s)	Intensity (%HRmax)
Week 1	COD training	Lateral hop step (30 s) Spider exercise (30 s)	30	3	15	70–89
	Reactive decision-making	Array: React to yellow (30 s) React to green (30 s) Double-leg react (slow/fast) (20 s)	30	3	15	70–89
	Movement transition	Foot fire split step (20 s) Quick steps forward (30 s) Crossover (forward/backward) (15 s)	30	3	15	70–89
Week 2	COD training	45- Degree hip turns (20 s) Spider exercise (30 s)	30	3	15	70–89
	Reactive decision-making	Reactive stability (left/right) (30 s) Double-leg react (slow/fast) (20 s) Array: React to red (30 s) React to green/yellow/red (30 s)	30	3	15	70–89
	Movement transition	Gait speed (forward/backward) (20 s) Foot fire staggered (20 s)	30	3	15	70–89
Week 3	COD training	Hip-turn jump (20 s) Spider exercise (30 s)	30	3	15	70–89
	Reactive decision-making	Reactive stability (left/right) (30 s) Double-leg react (slow/fast) (20 s) Array: React to red (30 s) React to green/yellow/red (30 s)	30	3	15	70–89
	Movement transition	Gait speed (forward/backward) (20 s) Foot fire staggered (20 s)	30	3	15	70–89
Week 4	COD training	45- Degree hip turns (20 s) Hip-turn jump (20 s) Spider exercise (30 s)	30	3	15	70–89
	Reactive decision-making	Array reaction foot fire (20 s) React to green/red (20 s) Foot fire go/no-go (forward/backward) (20 s)	30	3	15	70–89
	Movement transition	Stagger step (left/right) (20 s) Gait speed (forward/backward) (20 s)	30	3	15	70–89

Blocks 1–2 were performed without the ball; Block 3 was performed with dribbling. Rest indicates the planned inter-drill rest within each set. Intensity denotes the targeted training zone expressed as %HRmax.

**TABLE 3:** Training content for RLT.

Week	Type	Content	Duration (min)	Sets (n)	Inter-drill rest (s)	Intensity (%HRmax)
Week 1	COD training	Lateral slide Step-by-step ladder run	30	3	15	70–89
	Reactive decision-making	Quick forward run (small steps) Quick forward run with ladder exits Single-leg high-knee with ladder exits	30	3	15	70–89
	Movement transition	Quick stride (forward/backward) Fast forward run Cross-step (forward/backward)	30	3	15	70–89
Week 2	COD training	45-Degree hip slide Spider step (coach cue)	30	3	15	70–89
	Reactive decision-making	Single-leg to two-leg switch jump Crossed-legs slide Step forward/backward Side step forward/backward	30	3	15	70–89
	Movement transition	Z- shaped side steps Quick steps with legs apart (forward/backward)	30	3	15	70–89
Week 3	COD training	Hip hop Spider step	30	3	15	70–89
	Reactive decision-making	Single-leg balance jump (left/right) Two-foot bounce (slow/fast) Deceleration run Lateral quick steps with high knees	30	3	15	70–89
	Movement transition	Forward-frame movement Quick steps with legs apart (forward/backward)	30	3	15	70–89
Week 4	COD training	45- Degree hip slide Hip hop with forward foot movement Spider step	30	3	15	70–89
	Reactive decision-making	Forward step + lateral advance/retreat Lateral crossover Stop-and-go forward then lateral move	30	3	15	70–89
	Movement transition	Quick test steps (left + right) Forward-backward step speed	30	3	15	70–89

Blocks 1–2 were performed without the ball; Block 3 was performed with dribbling. Rest indicates the planned inter-drill rest within each set. Intensity denotes the targeted training zone expressed as %HRmax.

### 2.3 Intervention protocol

**FIGURE 1:**
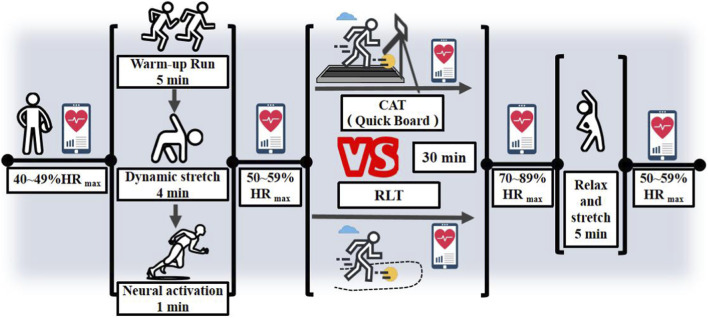
Experimental process diagram.

Both groups trained three times per week for 4 weeks (12 sessions in total). Each session lasted 45 min and targeted three agility components: change of direction, action transformation, and reaction and decision-making ability. A standardized session structure was implemented, consisting of a 10-min dynamic warm-up, a 30-min main training period, and a 5-min stretching and cool-down (Figure 1). Each session comprised three exercise blocks, with each block including 2–3 agility drills, 3–5 reactive drills, and 2–3 speed drills.

Throughout training, participants wore Firstbeat devices for real-time heart-rate monitoring to support adherence to the prescribed intensity and to ensure that training was broadly maintained within the target zone (70%–89% HRmax) during the main training period. Real-time monitoring focused on HR-related indicators (e.g., current HR/%HRmax and time accumulated within the target zone) for session supervision purposes (Bogdány et al., 2016; Conte et al., 2025). Based on real-time feedback, coaches adjusted work–rest timing when necessary to maintain the intended intensity while preserving movement quality. The exercise selection was basketball-specific: Blocks 1–2 emphasized non-ball movement tasks, whereas Block 3 incorporated ball-handling (dribble-based) drills. All training actions were finalized following expert consultation and are detailed in Tables 2, 3.

### 2.4 Testing procedures

Four domains were assessed: change of direction, action transformation, reaction and decision-making, and basketball-specific performance. All tests were administered before and after the intervention, scheduled at the same time and on the same day of the week to minimize diurnal variation. For all tests, the best performance at each time point was retained for analysis.

1. Action transformation ability: Footwork speed test: number of pad contacts (foot taps); CBST: completion time.
2. Change of direction ability: T-test of agility: completion time.
3. Reaction and decision-making ability: Choice reaction test: accuracy (%) and mean reaction time (s); CBST visual decision latency: mean reaction time (s) recorded within the CBST.
4. Basketball-specific performance: CBST performance time; CBST skill penalty time; CBST total completion time.

#### 2.4.1 Footwork speed test

Footwork speed was assessed using the Quick Board system (test-retest reliability: ICC = 0.89) (Galpin et al., 2008). The device connects to an iPad via Bluetooth and features five pressure-sensitive pads—upper-right, upper-left, lower-right, lower-left, and center—providing light-based visual stimuli and feedback.

**FIGURE 2:**
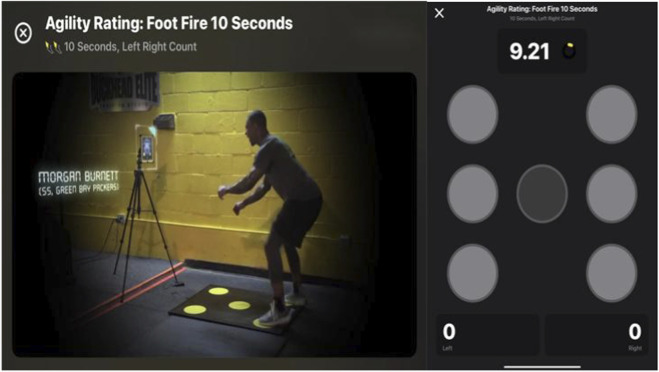
Footwork speed test.

Participants began with both feet in a neutral position, not contacting any pad. After a 5-s countdown on the iPad, the trial started by placing the right foot on the lower-right pad, followed by rapid alternating steps, starting with the left foot on the lower-left pad. Participants were instructed to complete the task as quickly as possible (Figure 2).

The iPad-based software automatically recorded the number of pad contacts completed within 10 s. Two trials were performed, separated by 60 s of rest, the higher reps was retained for analysis.

#### 2.4.2 Choice reaction test

**FIGURE 3:**
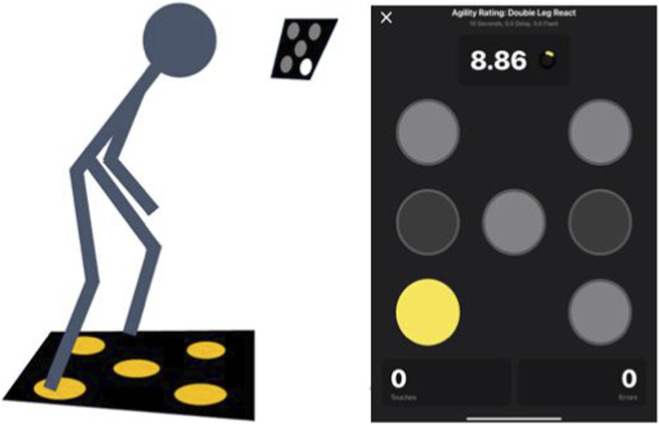
Choice reaction test.

The choice reaction test (ICC = 0.89) was assessed on the Quick Board (Galpin et al., 2008). Participants stood in a neutral stance with feet positioned between the left–right pads, avoiding contact with the center pad. After a 5-s countdown, a target light illuminated randomly. Participants struck the corresponding pad with the matching foot for left/right targets; the center target could be struck with either foot. After each successful contact, a new target appeared randomly, and participants returned to neutral before responding again. To minimize learning effects, the software generated random activation patterns (Figure 3).

Each 10-s trial recorded: (a) number of correct contacts, (b) number of errors, and (c) mean reaction time (s). Accuracy was calculated as correct/(correct + errors). Two trials were performed with 60 s of rest. The trial with the lower mean reaction time was retained for analysis. All outcomes (correct contacts, errors, mean reaction time, and accuracy) were derived from the retained trial.

#### 2.4.3 T-test

The T-test was used to assess multidirectional agility and speed, which are relevant to basketball-specific movement demands (Sugiyama et al., 2021; Morral-Yepes et al., 2022).

**FIGURE 4:**
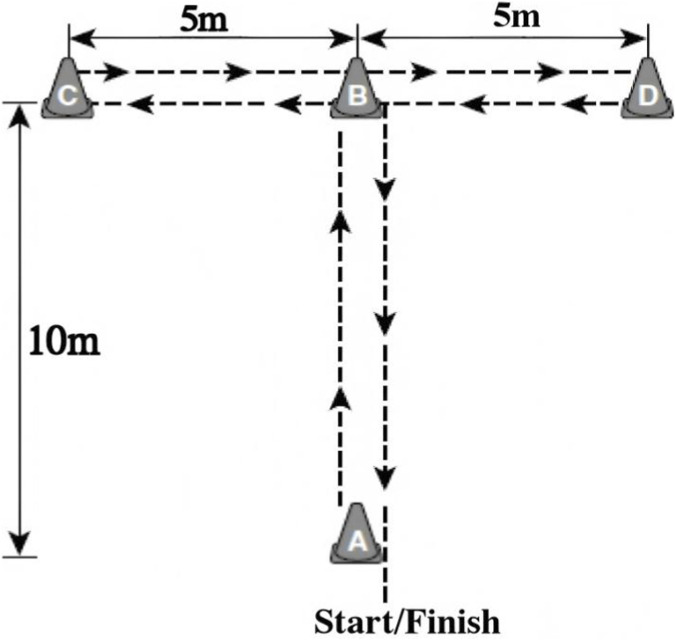
T-Test of agility.

Procedure: Participants began at cone A, sprinted to cone B, shuffled to cone C, moved across to cone D, shuffled back to cone B, and backpedaled to the starting point at cone A (Figure 4). Performance time (s) was recorded using Smart Speed timing gates (Fusion Sport, Coopers Plains, Australia). Each participant completed two trials, and the faster time was retained for analysis.

#### 2.4.4 Combined Basketball Skill Test (CBST)

The Combined Basketball Skill Test (CBST) (ICC across variables: 0.83–0.96) (Conte et al., 2019) was used to evaluate integrated performance in technical execution and decision-making. The protocol consisted of 12 trials, each separated by 60 s, initiated by a visual stimulus.

**FIGURE 5:**
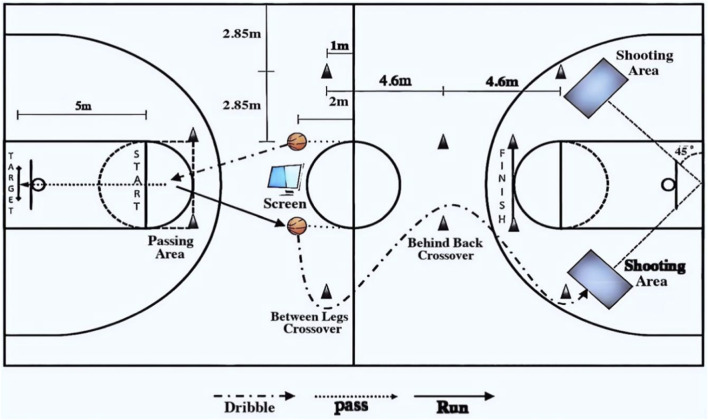
Combined basketball skill test scheme.

Procedure (Figure 5): Participants started behind the free-throw line, facing a display screen 5 m ahead. The test began when a visual cue appeared. Based on the symbol displayed—square = jump shot, circle = lay-up, triangle = reverse lay-up—players retrieved a basketball from one of two vertical posts (0.8 m high, located 2 m in front of the mid-court line; red = right hand, black = left hand). After retrieving the ball, participants dribbled to the passing zone and performed a bounce pass to a 5-m target (line contact allowed, crossing prohibited). They then retrieved a second ball, dribbled around a cone, and proceeded toward either sideline using the appropriate hand. Dribbling tasks included between-legs crossover, behind-the-back crossover, and spin dribbling (without switching hands). Finally, participants performed the designated shooting action and sprinted to the finish line at the top of the key.

Penalty scoring was based on error severity (Table 4). CBST outcome variables included: (a) completion time (sum of 12 trials), (b) penalty time (cumulative penalties), (c) performance time (completion time + penalty time), and (d) initiation reaction time (time from stimulus onset to movement initiation). Visual stimuli were generated using PowerPoint (Microsoft Corp., United States). Each cue was displayed for 10 s, followed by a 50-s countdown interval; each shooting action appeared twice in a randomized order. At each time point, participants completed two CBST sessions (each consisting of 12 trials) separated by a standardized rest period. The session with the lower performance time was retained for analysis, and all CBST outcomes were taken from the retained session.

**TABLE 4:** Penalty time associated to each error during the combined basketball skill test.

Action	Error	Penalty time (s)
Pass	Bull’s eye	−1
	Hit the ring (going in)	0
	Hit the ring (going out)	1
	Missed the ring	3
	Wrong passing area	5
	No bounce pass	5
Ball control	Correct dribble	0
	Wrong crossover	2
	Wrong hand	3
	Lost ball control	5
Shot	Scored jump shot	0
	Missed jump shot (hitting the ring)	2
	Missed jump shot (air-ball)	4
	Wrong area	4
	Score layup	0
	Missed layups (hitting the ring)	3
	Missed layups (air-ball)	5
	Layup with wrong hand	2
	Score reverse layup	0
	Missed reverse layups (hitting the ring)	2
	Missed reverse layups (air-ball)	4
	Reverse layup with wrong hand	2
Decision making	Correct ball (color)	0
	Wrong ball (color)	4
	Correct shot (shape)	0
	Wrong shot (shape)	4
Violation	Moved before the visual stimulus	3
	Turnover (travelling/dribble violation/out of bounds	5

### 2.5 Statistical analysis

Blinded statistical analyses were performed by a third researcher, unaware of experimental group assignments. We employed linear mixed-effects models (LMM) as the primary analytical approach to account for repeated measurements within participants and to accommodate unbalanced data structures and incomplete cases under a missing-at-random assumption (Bates et al., 2015). Compared with paired t-tests or repeated-measures ANOVA, LMM provide a flexible framework for longitudinal sport-performance data by explicitly modeling within-participant dependence and inter-individual heterogeneity while retaining all available observations for inference (Bates et al., 2015; Pinheiro and Bates, 2000). For outcomes assessed with two attempts, the best performance at each time point was retained prior to modeling (highest value for repetition- or accuracy-based outcomes; lowest value for time-based outcomes).

**FIGURE 6:**
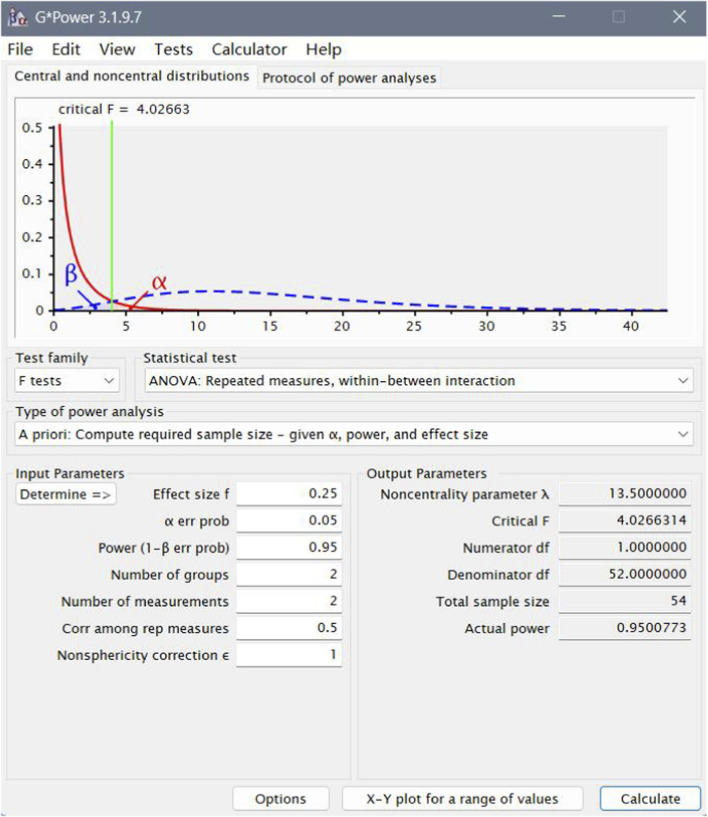
Detail of sample size calculation.

*A priori* sample size estimation was performed using G*Power software (version 3.1.9.7; Franz Faul, University of Kiel, Germany) (Ruxton and Neuhäuser, 2010), following established recommendations for power analysis in sports science (Beck, 2013; Atkinson and Nevill, 1998). Since G*Power does not directly support LMM, the design was approximated using a mixed-design ANOVA (repeated-measures, within-between interaction F-test) (Cook et al., 2002; Hopkins, 2000; West et al., 2022; Singer, 2003; Faul et al., 2007). A small-to-moderate effect size (Cohen’s f = 0.20) was specified based on conservative estimates reported in prior work (Batterham and Hopkins, 2006; Murphy et al., 2023; Mesquida et al., 2022). Based on these parameters (Figure 6), the minimum required sample size was 54 participants (Lakens, 2013). To enhance methodological rigor, improve statistical robustness, and reduce the risk of Type II error, the target sample size was increased to 64 (Ioannidis, 2005; Kaur and Stoltzfus, 2017). The final sample comprised 64 athletes (Centers = 12; Forwards = 26; Guards = 26).

For each outcome, the test metric was specified as the dependent variable. Fixed effects included Group (CAT vs. RLT), Position (center, forward, guard), and Time (pre, post), along with all two-way and three-way interactions. A random intercept for participant was included to account for within-participant dependence (Carey and Wang, 2001). Models were fitted using restricted maximum likelihood (REML) with the lme4 package. Degrees of freedom and p-values for fixed effects were obtained using Satterthwaite’s approximation as implemented in lmerTest (Bates et al., 2015). Post hoc simple-effects and pairwise comparisons were performed using model-estimated marginal means (emmeans), with Bonferroni adjustment for multiple testing.

Model assumptions were evaluated using residual diagnostics (Q–Q plots, residual histograms, and residuals-versus-fitted plots) to assess normality and homoscedasticity. Statistical significance was set at p < 0.05. Effect sizes were expressed as Cohen’s f derived from marginal *R* ^2^ values for fixed effects (*R* ^2^_m), with thresholds of 0.10 (small), 0.25 (medium), and 0.40 (large) (Cohen, 1988; Middel and Van Sonderen, 2002). All analyses were conducted in R (version 4.4.1; R Core Team, 2024).

## 3 Results

A total of 64 male basketball players completed the intervention and were randomly assigned to either the computerized agility training (CAT) group or the rope ladder training (RLT) group for a 4-week training program. Athletes completed four performance assessments before and after the intervention, yielding eight outcome variables: foot-tapping count from the foot-speed test; mean reaction time and accuracy from the choice-reaction test; completion time from the T-agility test; and performance time, skill penalty time, and completion time from the CBST. Each outcome was analyzed using data from 64 participants measured at two time points (n = 128 observations per outcome). Baseline comparisons revealed no significant between-group differences in any performance variables (all p > 0.05; Table 1), indicating well-balanced groups and strong comparability prior to the intervention.

Across the eight linear mixed-effects models, marginal *R* ^2^ values ranged from 0.013 to 0.356, indicating that fixed effects explained a modest proportion of outcome variance. In contrast, conditional *R* ^2^ values were consistently higher (0.415–0.954), reflecting substantial between-subject variability across performance measures. Effect sizes, expressed as Cohen’s f, ranged from small to large (0.115–0.744), depending on the outcome.

Diagnostic checks confirmed that all LMM satisfied model assumptions. Residual diagnostics indicated no meaningful violations of model assumptions. Visual inspection of Q–Q plots and residuals-versus-fitted plots suggested approximate normality and homoscedasticity, with no influential outliers detected. No data transformations were required, supporting the robustness of the subsequent analyses.

### 3.1 Agility

#### 3.1.1 Action transformation ability

Action transformation ability was assessed using the number of foot-taps recorded during the foot-speed test. Type III ANOVA (Satterthwaite) results (Table 6) revealed a significant main effect of Time (F_(1,58)_ = 33.56, p < 0.001), indicating an overall increase in foot-speed performance from pre-to post-test. A significant main effect of Position was also observed (F_(2,58)_ = 14.59, p < 0.001), indicating clear positional differences in foot-speed performance, with guards showing the highest estimated means.

**FIGURE 7:**
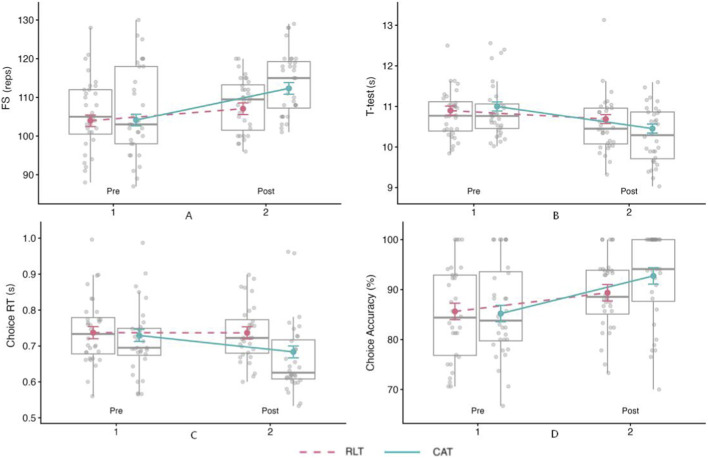
Pre–post changes in agility-related performance between the Computerized Agility Training (CAT) and Rope Ladder Training (RLT) groups. **(A)** Foot speed (FS). **(B)** T-test agility time. **(C)** Choice reaction time (Choice RT). **(D)** Choice accuracy. Boxplots show individual variability, and lines represent group mean trends across time points.

Importantly, a significant Group × Time interaction emerged (F_(1,58)_ = 6.74, p = 0.012, β = 1.27 ± 0.49, t_(58)_ = 2.59, 95% CI [0.29, 2.25]), indicating that pre–post changes differed between the CAT and RLT groups. No other two-way or three-way interactions reached statistical significance (all p > 0.05). Consistent with the descriptive results (Table 5) and Figure 7A, the CAT group exhibited a larger improvement (7.0%) than the RLT group (2.4%), with a steeper pre–post trajectory for CAT.

**TABLE 5:** Descriptive statistics and pre-to-post changes in basketball performance variables for the CAT and RLT groups.

Variable	CAT (n = 32)			RLT (n = 32)			CAT vs. RLT p-value (pre)
	Pre	Post	Δ (%Δ)	Pre	Post	Δ (%Δ)	
Foot speed test (reps)	106.28 (2.07)	113.69 (1.45)	7.4 (7.0%)	105.62 (1.66)	108.12 (1.23)	2.5 (2.4%)	0.81
Choice react time (s)	0.71 (0.02)	0.67 (0.02)	−0.05 (−6.9%)	0.74 (0.02)	0.74 (0.01)	−0.006 (−0.7%)	0.28
Choice accuracy (%)	0.86 (0.02)	0.93 (0.02)	0.07 (7.5%)	0.85 (0.02)	0.89 (0.01)	0.04 (4.8%)	0.74
T- test (s)	10.89 (0.12)	10.31 (0.12)	−0.59 (−5.4%)	10.81 (0.10)	10.57 (0.13)	−0.25 (−2.3%)	0.61
CBST react time (s)	0.68 (0.01)	0.62 (0.01)	−0.07 (−9.8%)	0.70 (0.01)	0.69 (0.01)	−0.01 (−1.4%)	0.40
CBST performance time (s)	244.66 (3.72)	239.53 (4.09)	−5.13 (−2.1%)	244.59 (2.94)	243.84 (2.70)	−0.75 (−0.3%)	0.99
CBST penalty time (s)	44.66 (2.11)	44.38 (2.29)	−0.28 (−0.6%)	45.66 (1.50)	46.12 (1.40)	0.47 (1.0%)	0.70
CBST completion time (s)	201.69 (2.73)	197.12 (2.76)	−4.56 (−2.3%)	197.75 (2.63)	196.78 (2.59)	−0.97 (−0.5%)	0.30

Values are presented as mean ± standard error (SE).

Δ indicates the absolute change from pre-to post-test, and (%Δ) represents the percentage change relative to pre-test values.

*p*-values were obtained from independent t-tests comparing baseline (pre-test) values between groups. All tests were performed using two-tailed significance thresholds (p < .05).

Units: reps, repetitions; s, seconds; %, accuracy percentage. Improvements are indicated by positive Δ for performance-enhancing metrics (e.g., repetitions, accuracy) and negative Δ for time-based metrics (e.g., reaction time, T-test, CBST).

Taken together, these findings suggest that computerized agility training was associated with greater gains in action transformation ability than rope ladder training in basketball athletes.

#### 3.1.2 Change of direction (COD) ability

COD was evaluated using T-test completion time. As shown in Table 5, both groups improved following training. Type III ANOVA (Satterthwaite) results (Table 6) indicated significant main effects of Time (F_(1,58)_ = 26.56, p < 0.001, β = 0.19 ± 0.04, t_(58)_ = 5.15, 95% CI [0.12, 0.26]) and Position (F_(2,58)_ = 11.88, p < 0.001), indicates a reduction in completion time from pre-to post-test with persistent positional differences. Post hoc comparisons indicated that guards demonstrated shorter completion times than forwards (β = −0.34 ± 0.09, p < 0.001, t_(58)_ = −3.67, 95% CI [−0.52, −0.15]) and centers (β = −0.20 ± 0.09, p = 0.035, t_(58)_ = −2.16, 95% CI [−0.38, −0.01]).

**TABLE 6:** Linear mixed-effects model results for agility and basketball-specific performance outcomes.

Model (p-value)	Group	Time	Position	Group × Time	Group × Position	Time × Position	Group × Time × Position	Marginal *R* ^2^ (R^2^m)	Conditional *R* ^2^ (R^2^c)	Cohen’s f (overall)
Foot speed test	0.157	<0.001	<0.001	0.012	0.666	0.100	0.844	0.356	0.728	0.744
Choice react time	0.180	0.004	0.163	0.005	0.055	0.305	0.734	0.144	0.828	0.41
Choice accuracy	0.433	<0.001	0.475	0.173	0.099	0.372	0.086	0.183	0.954	0.473
T-test Time	0.636	<0.001	<0.001	0.026	0.144	0.359	0.774	0.152	0.415	0.423
CBST react time	0.083	<0.001	0.546	<0.001	0.126	0.629	0.909	0.271	0.709	0.61
CBST performance time	0.813	<0.001	<0.001	0.021	0.041	0.010	0.498	0.205	0.778	0.508
CBST penalty Time	0.589	0.956	0.016	0.845	0.809	0.714	0.004	0.181	0.944	0.47
CBST completion time	0.277	<0.001	0.002	<0.001	0.192	0.003	0.195	0.013	0.933	0.115

Each LMM, included Group (CAT, vs. RLT), Time (Pre vs. Post), and Position (Guard, Forward, Center) as fixed factors with random intercepts for participants.

Values represent p-values from Linear Mixed-Effects Models. Significance levels (p-values) were obtained from Type III F-tests (df_1_ = 1–2, df_2_ = 58). Marginal *R* ^2^ represents the variance explained by fixed effects, and Conditional *R* ^2^ includes both fixed and random effects (Nakagawa and Schielzeth, 2013).

Overall Cohen’s f was calculated from R^2^m using: $f=\sqrt{R^{2}m÷\left(1‐R^{2}m\right)}$ ). According to Cohen, (1988), f = 0.10, 0.25, and 0.40 indicate small, medium, and large effects, respectively. Cohen’s f ranged from 0.115 to 0.744, indicating small-to-large overall effect sizes across models.

A significant Group × Time interaction was also observed (F_(1,58)_ = 5.22, p = 0.026, β = −0.08 ± 0.04, t_(58)_ = −2.29, 95% CI [−0.16, −0.01]), indicating that pre–post changes in completion time differed between groups, with CAT showing a larger reduction than RLT. No other interaction terms reached significance (all p > 0.05). As illustrated in Figure 7B, both trajectories declined over time (lower = better), with a steeper decrease in the CAT group, consistent with the descriptive changes in Table 5.

Overall, these results indicate significant effects of training progression and playing position on COD performance, with CAT associated with greater improvements than RLT.

#### 3.1.3 Reaction and decision-making ability

Reaction and decision-making ability was evaluated through reaction time and decision accuracy.

**FIGURE 8:**
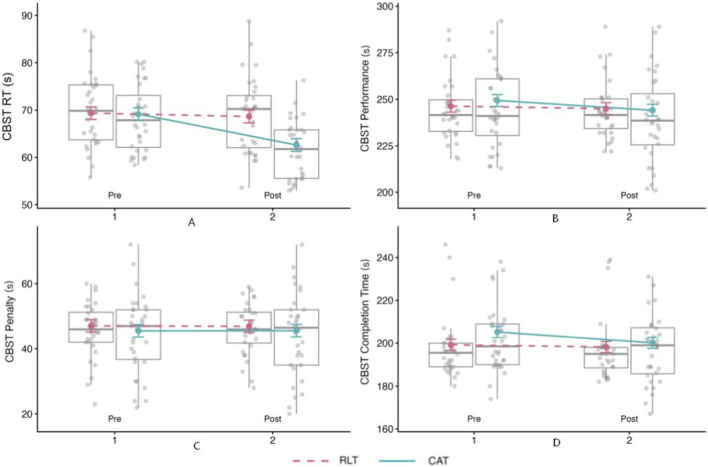
Pre–post differences in four CBST performance indicators for the CAT and RLT groups. **(A)** CBST reaction time (RT). **(B)** CBST performance score. **(C)** CBST penalty time. **(D)** CBST completion time. Boxplots show individual data, and lines indicate group mean trends across time points.

To quantify reaction speed, we analyzed both the choice reaction test reaction time (ChoiceRT) and the visual-stimulus response time embedded within the CBST (CBSTRT). As shown in Table 5, the CAT group exhibited larger pre–post reductions in reaction time (−6.9% and −9.8%, respectively) than the RLT group (−0.7% and −1.4%). Type III ANOVA (Satterthwaite) indicated significant main effects of Time for both reaction-time outcomes (ChoiceRT: F_(1,58)_ = 9.06, p = 0.004; CBSTRT: F_(1,58)_ = 25.18, p < 0.001) and significant Group × Time interactions (ChoiceRT: F_(1,58)_ = 8.70, p = 0.005; CBSTRT: F_(1,58)_ = 16.11, p < 0.001). Consistent with the parameter estimates and the trajectories in Figures 7C, 8A, these results indicate that pre–post changes in reaction time differed between groups, with CAT showing a steeper reduction than RLT (lower = better). No other interaction terms reached significance (all p >0.05).

Decision accuracy was assessed via choice-reaction accuracy (ChoiceAcc). Table 5 shows an improvement in both groups (CAT: +7.5%; RLT: +4.8%). The LMM revealed a significant main effect of Time (F_(1,58)_ = 17.12, p < 0.001, β = −0.03 ± 0.01, t_(58)_ = −4.13, 95% CI [−0.04, −0.01]), indicating overall increases in accuracy from pre-to post-test. However, the Group × Time interaction was not statistically significant (F_(1,58)_ = 1.91, p = 0.173, β = 0.01 ± 0.01, t_(58)_ = 1.38, 95% CI [0.00, 0.02]), providing no clear evidence that accuracy changes differed between CAT and RLT. The Group × Time × Position interaction showed a trend toward significance (F_(2, 58)_ = 2.56, p = 0.086), suggesting that position-dependent responses may warrant further investigation. As illustrated in Figure 7D, the CAT group (blue line) demonstrated more pronounced accuracy gains.

Collectively, these findings support that CAT elicited greater improvements than RLT in reaction speed (ChoiceRT and CBSTRT), whereas decision accuracy improved over time with no statistically significant between-group difference in the pre–post change.

### 3.2 Basketball-specific skill performance

The Combined Basketball Skill Test was employed to assess players’ technical execution and decision-making ability under time pressure. Performance time, defined as the sum of completion time and penalty time, reflects the integrated efficiency of speed and accuracy in basketball-specific skill execution.

#### 3.2.1 CBST performance time

CBST performance time decreased from pre-to post-test, with a larger reduction observed in the CAT group (−2.1%) than in the RLT group (−0.3%) (Table 5).

LMM results (Table 6) showed significant main effects of Time (F_(1,58)_ = 15.55, p < 0.001) and Position (F_(2,58)_ = 7.86, p < 0.001), indicating overall improvements in performance time alongside persistent positional differences. Significant Group × Time (F_(1, 58)_ = 5.66, p = 0.021), Group × Position (F_(2, 58)_ = 3.39, p = 0.041), and Time × Position interactions (F_(2, 58)_ = 5.01, p = 0.010) were also observed, whereas the Group main effect (p = 0.813) and the three-way interaction (p = 0.498) were not significant.

Consistent with these results, fixed-effect estimates indicated a significant pre–post improvement in performance time (β = 1.64 ± 0.42, t_(58)_ = 3.95, p < 0.001, 95% CI [0.81, 2.47]) and a significant Group × Time interaction (β = −0.99 ± 0.42, t_(58)_ = −2.38, p = 0.021, 95% CI [−1.82, −0.16]), demonstrating that the magnitude of improvement differed between groups. As illustrated in Figure 8B, the CAT group showed a steeper pre–post reduction in performance time than the RLT group.

Overall, these findings indicate that CAT was associated with greater improvements in basketball-specific technical performance than RLT, supporting the utility of computerized agility training for enhancing complex, game-relevant skill execution.

#### 3.2.2 CBST penalty time

The LMM results for penalty time showed that only the Position main effect was significant (F_(2,58)_ = 4.47, p = 0.016), whereas Group (p = 0.589), Time (p = 0.956), and Group × Time (p = 0.845) were not significant. Notably, however, a significant Group × Time × Position interaction emerged (F_(2,58)_ = 5.95, p = 0.004). Using guards in the RLT group at pre-test as the reference category, model estimates suggested that CAT led to a significant reduction in penalty time for centers (β = −1.07 ± 0.32, p = 0.002, t_(58)_ = −3.33, 95% CI [−1.71, −0.43]), whereas no meaningful change was observed for forwards (β = −0.01 ± 0.32, p = 0.976, t_(58)_ = −0.03, 95% CI [−0.65, 0.63]) or guards.

Consistent with Figure 8C, trajectories were largely flat at the group level, suggesting that error-reduction benefits were selective rather than generalized across positions.

#### 3.2.3 CBST completion time

CBST completion time (s) reflects the speed of executing the CBST action sequence (excluding error-related penalties).

Type III ANOVA (Satterthwaite) from the LMM indicated significant main effects of Time (F_(1,58)_ = 29.21, p < 0.001) and Position (F_(2,58)_ = 7.14, p = 0.002), while Group was not significant (F_(1,58)_ = 1.20, p = 0.277), indicating overall pre–post improvements alongside positional differences. Critically, a significant Group × Time interaction was observed (F_(1,58)_ = 13.13, p < 0.001, β = −1.02 ± 0.28, t_(58)_ = −3.62, 95% CI [−1.59, −0.46]), demonstrating that completion time decreased more in the CAT group than in the RLT group (Table 5; Figure 8D). A significant Time × Position interaction emerged (F_(2,58)_ = 6.51, p = 0.003), with the largest improvement in centers (β = −1.29 ± 0.37, p < 0.001, t_(58)_ = −3.50, 95% CI [−2.03, −0.55]), whereas changes in forwards (β = 0.65 ± 0.37, p = 0.084, t_(58)_ = 1.76, 95% CI [−0.09, 1.39]) were smaller and did not reach statistical significance. The Group × Position and three-way interactions were not significant.

Taken together, the CBST results revealed a consistent advantage of computerized agility training over rope ladder training for basketball-specific skill performance. Across CBST outcomes, CAT was associated with larger pre–post improvements in performance-related timing measures, as reflected by significant Group × Time interactions for performance time and completion time. In contrast, penalty time did not exhibit a uniform training effect at the group level, but instead showed a significant Group × Time × Position interaction, indicating position-dependent changes. Collectively, these findings suggest that the primary benefits of CAT on CBST performance were driven by faster task execution, with more selective effects on error-related costs.

## 4 Discussion

This 4-week randomized trial demonstrated that computerized agility training (CAT) produced greater improvements than rope ladder training (RLT) in male basketball players across multiple domains: action-transformation speed, change-of-direction performance, reaction and decision-making ability, and basketball-specific skill execution. The CAT implemented via the QuickBoard to deliver unpredictable visual stimuli and response-driven tasks, was more closely aligned with the dynamic, time-pressured demands of competitive play. It produced superior gains in visuomotor coordination, perceptual–cognitive processing, and functional agility. Compared with RLT’s fixed footwork patterns, CAT’s instant feedback and stimulus–response coupling likely engaged the neuromuscular system more effectively, thereby enhancing locomotor efficiency and on-court performance.

The study also identified marked position-specific differences in training responsiveness. In the CBST, centers showed a significantly greater reduction in penalty time than guards and forwards, suggesting that players with lower baseline agility (e.g., centers) may have greater scope for improvement under the same training stimulus. Consistent with previous research, guards typically outperform forwards and centers in agility- and speed-based assessments, reflecting position-specific locomotor and perceptual–motor demands. These findings underscore the importance of individualizing training prescriptions by considering positional requirements and initial capabilities when designing agility-oriented programs, rather than adopting a one-size-fits-all approach in basketball conditioning programs.

Substantial inter-individual variability in training responses was also observed. Although group-level trends were positive, the magnitude of improvement varied widely across athletes. By including random intercepts and random slopes in the linear mixed-effects models, we captured individual trajectories and obtained inferences that more closely reflect real-world training contexts. This modelling approach explicitly represents the heterogeneity of training responses, allowing both baseline performance and rates of change to be incorporated within the analytic framework. Recognizing that identical training stimuli can elicit divergent adaptations across athletes further underscores the importance of personalized program design and outcome evaluation. In line with current evidence on agility development in basketball, these findings provide useful guidance for future individualized training prescriptions and monitoring strategies.

### 4.1 Comparison with prior literature

Our findings are consistent with contemporary research on agility development, indicating that QuickBoard-based computerized agility training (CAT) leads to substantial improvements in reaction time, decision accuracy, and change-of-direction performance, with gains exceeding those typically reported following traditional rope ladder training (RLT) (Jorgensen et al., 2013; Galpin et al., 2008; Engeroff et al., 2018; Engeroff et al., 2021). Although studies derived QuickBoard specifically in basketball populations remain limited, the present results extend previous findings reported by Collins (2014) and Engeroff et al. (2019), who demonstrated that stimulus-driven agility training enhances visuomotor integration by coupling visual information with rapid motor responses, thereby facilitating more effective agility adaptations across sport contexts.

In contrast, improvements associated with RLT appear to be largely confined to rhythm, step frequency, and basic coordination, with relatively limited transfer to reaction-time and decision-making performance in open-skill contexts (Afonso et al., 2020). Prior studies have similarly reported non-significant or small effects of ladder-based drills on agility and dribble-related dynamic tasks (Padrón-Cabo et al., 2020), consistent with our observation that the RLT group showed minimal changes in reaction time and decision accuracy. Collectively, these findings underscore a key limitation of RLT: its fixed, preprogrammed movement patterns do not replicate the random, time-pressured stimuli characteristic of competitive play. Although recent dual-task ladder protocols can elicit modest improvements in cognition–action coupling (Castillo de Lima et al., 2023), their effects appear weaker than those fully stimulus-driven paradigms. In our study, the randomized visual cues embedded in CAT likely facilitated neural and perceptual–cognitive adaptations that support faster perceptual–decision responses, offering a plausible explanation for CAT’s superior effects.

The superior CBST performance observed in the CAT group is consistent with the broader advantages of open-skill agility training, which provides higher ecological validity and cognitive load and is thought to preferentially engage neural systems involved in perceptual–motor integration and decision-making under time pressure. Such training enhances visuomotor coupling and decision-making under game-realistic conditions, whereas these mechanisms are comparatively under-recruited during ladder drills that rely on fixed and preplanned movement sequences.

Overall, this study provides, to our knowledge, the first head-to-head comparison of CAT and RLT in a basketball-specific context. While both interventions produced measurable improvements, CAT yielded superior enhancements in both the magnitude and breadth of performance outcomes. These findings support the view that stimulus–response–driven training models offer greater efficacy than repetitive footwork paradigms and emphasize that agility development in basketball should integrate the full input–perception–decision–action chain within ecologically valid, game-representative environments.

### 4.2 Mechanistic interpretation

The superior performance gains observed following computerized agility training (CAT), compared with rope ladder training (RLT), are likely attributable to differences in perceptual–cognitive demands and neuro-mechanical engagement. CAT emphasizes visuomotor coupling and stimulus-driven decision-making, which more closely reflect the requirements of reactive agility in open-skill sports such as basketball (Hassan et al., 2022; Wang et al., 2024; Paul et al., 2016; Zhang et al., 2025; Li et al., 2024; Silvestri et al., 2023). In contrast, RLT primarily targets rhythmic coordination and step-frequency regulation, resulting mainly in improvements in foundational movement coordination rather than decision-dependent agility (Afonso et al., 2020; Paul et al., 2016).

Delivered via the QuickBoard platform, CAT exposes athletes to unpredictable visual stimuli that require continuous perception–action coupling and rapid response selection (Wang et al., 2024; Zhang et al., 2025; Steff et al., 2024; Hassan, 2025). Such training is proposed to preferentially engage higher-order perceptual–motor control systems, thereby facilitating sensorimotor plasticity and more efficient movement adaptation under time pressure (Paul et al., 2016; Zhang et al., 2025; Li et al., 2024; Panchuk et al., 2018; Gou and Li, 2023; Mangine et al., 2014; Lucia et al., 2023). By comparison, RLT relies on repetitive, preprogrammed movement patterns that impose relatively low perceptual–cognitive demands and are more strongly associated with automatized motor control processes (Sun et al., 2025; Afonso et al., 2020). Consequently, CAT more effectively promotes reactive agility and basketball-specific performance outcomes that depend on rapid decision-making and movement adjustment.

### 4.3 Training period and intensity

The four-week intervention adopted in the present study aligns with commonly used short mesocycles in the agility-training literature and may be sufficient to elicit short-term improvements in relevant performance outcomes. Previous investigations using computerized agility platforms have reported improvements in foot-speed performance, reaction time, and postural control following four-week programs delivered three times per week with session durations comparable to those used here (Engeroff et al., 2019; Collins, 2014), suggesting that short-term, high-frequency agility training can yield measurable adaptations on test-based indicators.

With respect to rope ladder training, prior research has suggested an effective training window of approximately 4–8 weeks, with shorter interventions often yielding limited effects and longer programs potentially exhibiting diminishing returns due to skill saturation and accumulated fatigue (Afonso et al., 2020). Consistent with this view, Padrón-Cabo et al. (2020) reported minimal changes in agility outcomes following 6 weeks of isolated ladder-based drills, indicating that repetitive footwork training may require integration with reactive or decision-based elements to sustain performance gains over time.

Regarding training intensity, both CAT and RLT were prescribed at a moderate-to-vigorous intensity (70%–89% HRmax), which is generally considered sufficient to stimulate neuromuscular and perceptual demands while preserving movement quality (Afonso et al., 2020). By standardizing training frequency, planned session structure, and the intended intensity prescription, and by monitoring heart rate in real time to promote adherence to the target zone (70%–89% HRmax), we aimed to reduce confounding by training load. Nevertheless, heart-rate–derived internal-load indices were not exported and statistically summarized, and external training load was not comprehensively quantified; therefore, residual load-related confounding cannot be fully excluded. Neuroadaptive responses to agility-oriented training are often observed within the first 3 weeks and tend to stabilize thereafter (Engeroff et al., 2019). Accordingly, the present four-week mesocycle was designed to capture the early phase of adaptation while minimizing fatigue-related interference.

Overall, the present findings indicate that a 4-week program can elicit meaningful short-term improvements under standardized frequency, session structure, and intended intensity conditions; nevertheless, longer interventions and match-derived outcomes are required to determine whether these gains are optimized, retained, and translated to competitive match performance.

### 4.4 Position difference

Our data reveal position-dependent improvements in agility outcomes (guards, forwards, and centers), reflecting the combined influence of role-specific functional demands and baseline agility (Pradana et al., 2020; Liu, 2023). Guards showed smaller gains, consistent with a ceiling effect due to their already superior agility and reaction capabilities. Their regular exposure to high-frequency decision-making and frequent change-of-direction tasks likely maintains their sensorimotor coordination at a high level (Jin et al., 2023). Forwards showed moderate improvements, particularly in postural control and decision speed, likely reflecting their hybrid functional role requiring both explosiveness and stability. Centers demonstrated the largest gains, especially in reaction time and change-of-direction performance, aligning with previous reports indicating greater adaptive potential among athletes with lower baseline agility (Afonso et al., 2020).

Taken together, these position-specific responses support the task-specificity principle of agility training and highlight the importance of considering positional roles when interpreting training effects. Rather than adopting uniform training prescriptions, agility development programs may benefit from adjusting the emphasis of perceptual, cognitive, and postural demands according to positional requirements.

### 4.5 Combination of basketball performance

Taken together, the present findings indicate that agility training yields the greatest performance benefits when improvements in speed and coordination are functionally integrated with perceptual–cognitive demands. The superior outcomes observed following computerized agility training, particularly in tasks such as the Combined Basketball Skill Test, suggest that enhancements in reaction speed and change-of-direction ability are more likely to translate into effective basketball-specific performance when training emphasizes perception–action coupling under time pressure. These results highlight the importance of designing agility interventions that extend beyond isolated footwork speed and instead promote the coordinated execution of decision-making and movement within game-representative contexts.

## 5 Methodological considerations

To reduce inter-individual variability and strengthen internal validity, several methodological controls were incorporated into the study design. All participants completed a 1-week familiarization period prior to baseline testing, and training intensity was monitored using Firstbeat heart-rate sensors to support adherence to the prescribed target zone (70%–89% HRmax) during sessions. Participants were randomly assigned to the CAT and RLT groups, resulting in comparable baseline characteristics. Both groups followed identical training frequency, session structure, and intended intensity prescription, which was intended to reduce (but not eliminate) potential confounding due to unequal training exposure.

Linear mixed-effects models were employed to retain the full dataset and account for within-subject variability, practice-related effects, and baseline heterogeneity. Participant identity was included as a random intercept, and all models demonstrated satisfactory convergence, supporting reliable estimation of main and interaction effects.

In addition, session scheduling, load progression, and inter-set recovery were standardized across groups. The use of a consistent heart-rate zone, fixed training timetable, and controlled rest intervals was intended to limit fatigue-related variability and enhance the reliability of the observed training effects.

## 6 Limitations

This study has several limitations. First, the sample consisted exclusively of male collegiate basketball players, which may limit the generalizability of the findings to female athletes, other age groups, and higher competitive levels (e.g., elite or professional players).

Second, although the intervention sessions were designed to be comparable across groups (i.e., identical frequency, session duration, and planned structure) and heart rate was monitored in real time to support adherence to the prescribed intensity (with training broadly maintained within 70%–89% HRmax), we did not export or statistically summarize heart-rate–derived internal-load indices, nor did we comprehensively quantify external training load (e.g., movement counts, accelerations/decelerations, or change-of-direction volume) or collect session-RPE. Therefore, residual confounding related to total training load cannot be fully excluded.

Third, the outcome measures were primarily performance-based (e.g., change-of-direction ability, reaction time, and the Combined Basketball Skill Test) and did not include direct neurophysiological or higher-order cognitive assessments; thus, mechanistic inferences remain indirect.

Fourth, the relatively small number of centers (n = 6) likely reduced the precision of position-specific estimates, although this limitation does not materially change the interpretation of the primary outcomes.

Finally, while the four-week intervention aligns with practical training constraints, its duration precluded evaluation of longer-term adaptations, retention, and transfer to competitive match play. In addition, a novelty- or motivation-related effect associated with the computerized training modality cannot be ruled out and may have contributed to short-term improvements.

Future studies should employ longer interventions with comprehensive external-load monitoring, incorporate standardized internal-load indices (e.g., session-RPE and summarized heart-rate metrics), and use ecologically valid game-based scenarios to confirm durability and applied relevance.

## 7 Conclusion

The present study demonstrates that rope ladder training (as implemented here) is did not elicit comparable improvements in basketball-specific agility when compared with stimulus-driven computerized agility training. Under a rigorously standardized four-week protocol, computerized agility training produced broader and more substantial improvements across agility and basketball-specific skill execution than traditional ladder-based drills.

These results raise a critical question for contemporary training practice: to what extent are widely adopted methods truly effective for key game-related abilities? Our findings indicate that agility development in basketball is not solely a function of repetitive footwork speed but rather depends on the effective integration of perception, decision-making, and movement execution under time pressure. Training responses also varied according to positional role and baseline agility, highlighting the limitations of uniform conditioning strategies and reinforcing the need for individualized, context-specific agility programs.

From both mechanistic and applied perspectives, computerized agility training appears to enhance performance by strengthening perception–action coupling through unpredictable stimuli and real-time feedback, thereby promoting more effective transfer to game-representative situations. Practically, incorporating computerized agility training as a complement to traditional ladder-based methods may help optimize decision-making efficiency, movement transitions, and postural control across different positional profiles in competitive basketball.

## Data Availability

The raw data supporting the conclusions of this article will be made available by the authors, without undue reservation.
